# Supplementary material for: Establishment of a CRISPR/Cas9-Mediated Efficient Knockout System of Trichoderma hamatum T21 and Pigment Synthesis PKS Gene Knockout
Source: J Fungi (Basel). 2023 May 19;9(5):595. doi: 10.3390/jof9050595 (PMC10219154; doi:10.3390/jof9050595)
Supplement: Supplementary file 1 [file jof-09-00595-s001.zip › jof-2378322-supplementary.pdf]

Supporting Information

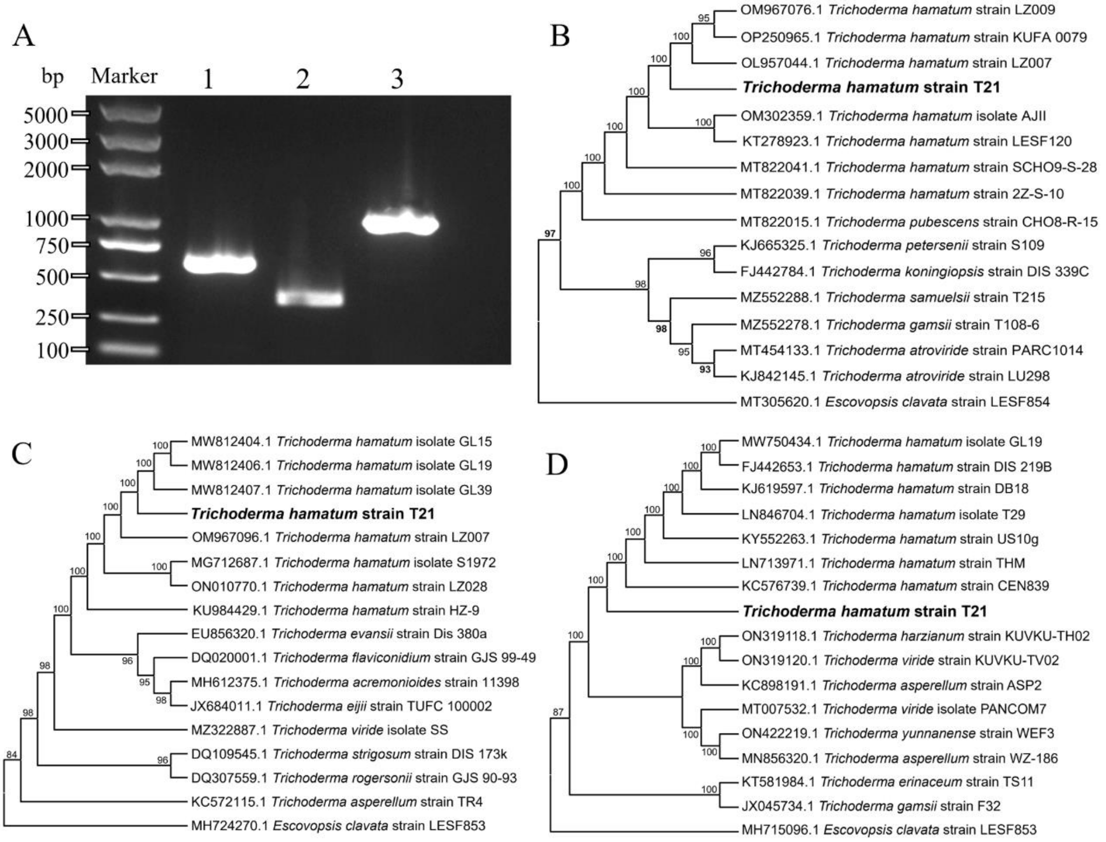

Figure S1. Molecular identification and phylogenetic analysis of *T. hamatum* T21. (**A**) PCR products of the ITS (lane 1), *tef1α* (lane 2), *rpb2* (lane 3) gene of *T. hamatum* T21. Phylogenetic tree of *T. hamatum* T21 based on ITS (**B**), *tef1α* (**C**), and *rpb2* (**D**) sequences.

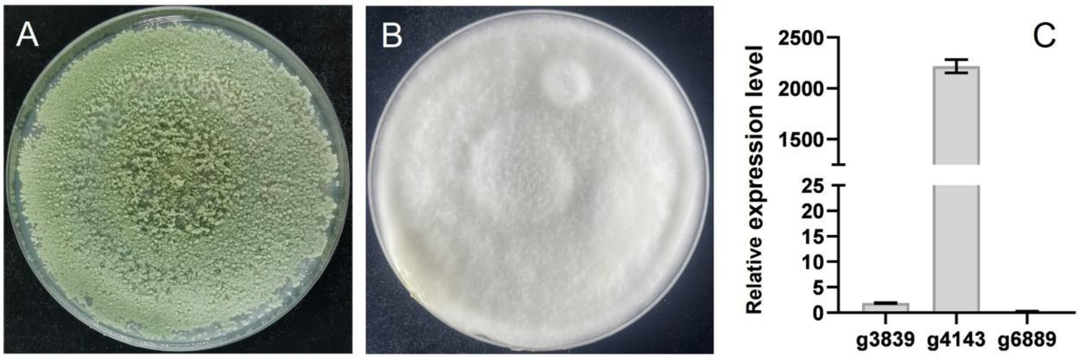

Figure S2. Phenotypic characteristics of *T. hamatun* T21 on different mediums and analysis of pigment candidate genes.

(**A**). PDA medium. (**B**) MOF medium. (**C**) A relative expression values of the three pigment candidate genes in two mediums. The *g3839*, *g4143*, and *g6889* genes were highly homologous to the pigment genes of *M. robergii* and were identified as candidate genes.

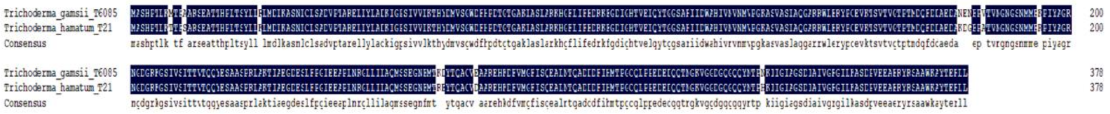

Figure S3. The sequence alignment of the orotidine 5'-phosphate decarboxylase in *T. gamsii* and *T. hamatun*.

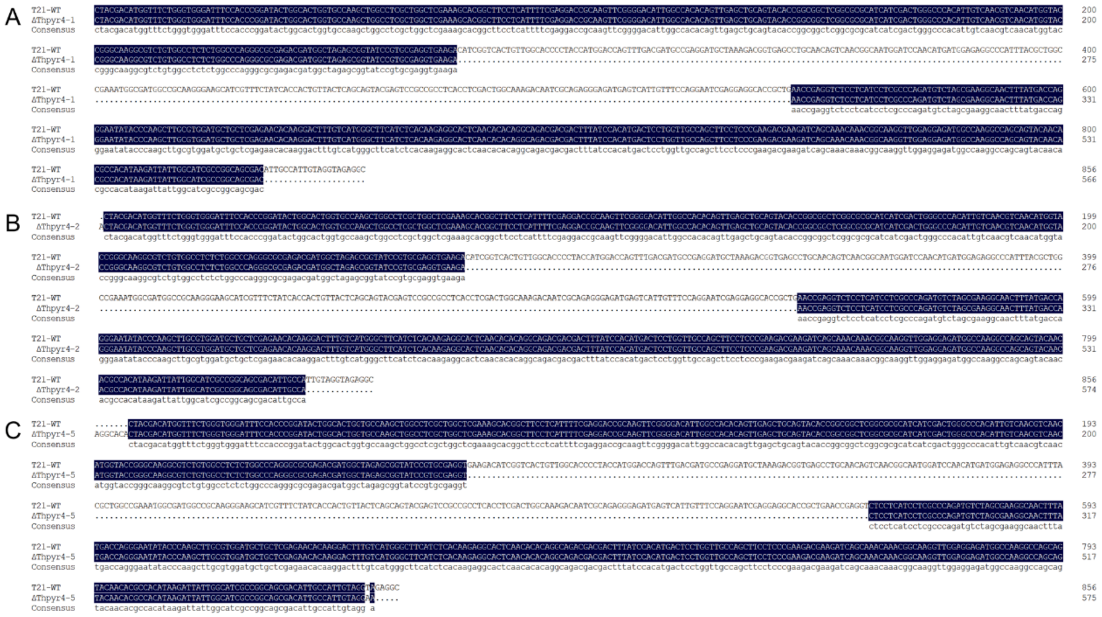

Figure S4. Comparison of knockout mutant target sequences with T21 wild-type sequences.

(**A**) Represents Δ*Thpyr4*-1. (**B**) Represents Δ*Thpyr4*-2. (**C**) Represents Δ*Thpyr4*-5 and corresponds to lanes 1, 2, and 5 in Figure 5-D respectively.

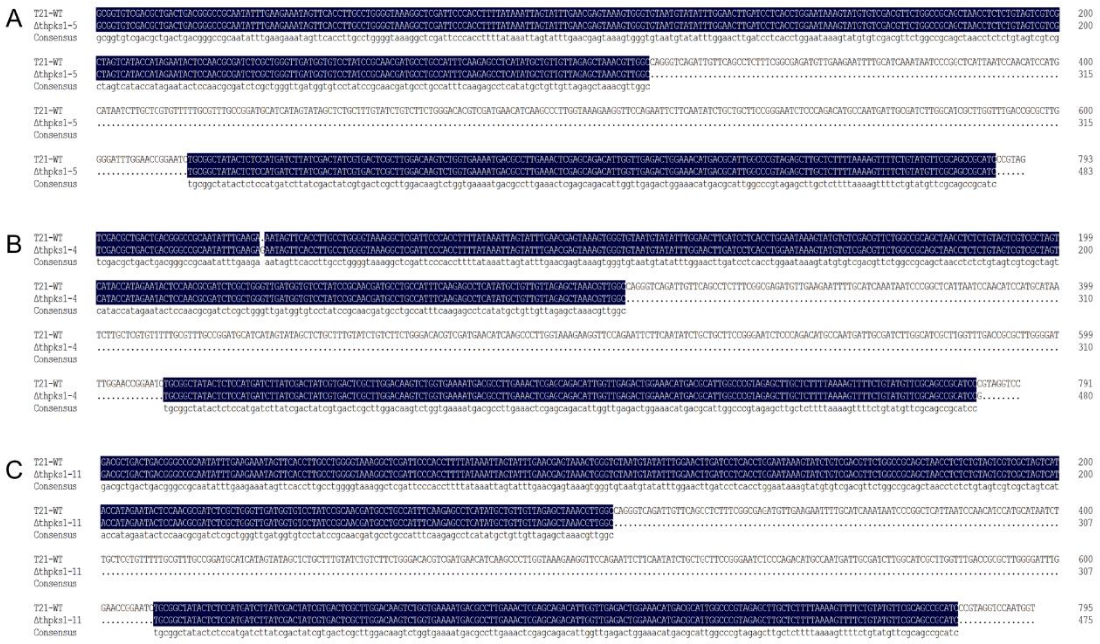

Figure S5. Comparison of knockout mutant target sequences with T21 wild-type sequences.

(**A**) Represents Δ*Thpks1*-5 and corresponds to Figure 6 (E-lane 5). (**B**) Represents Δ*Thpks1*-4. (**C**) Represents Δ*Thpks1*-11 and corresponds to lanes 4 and 11 in Figure 7-B respectively.

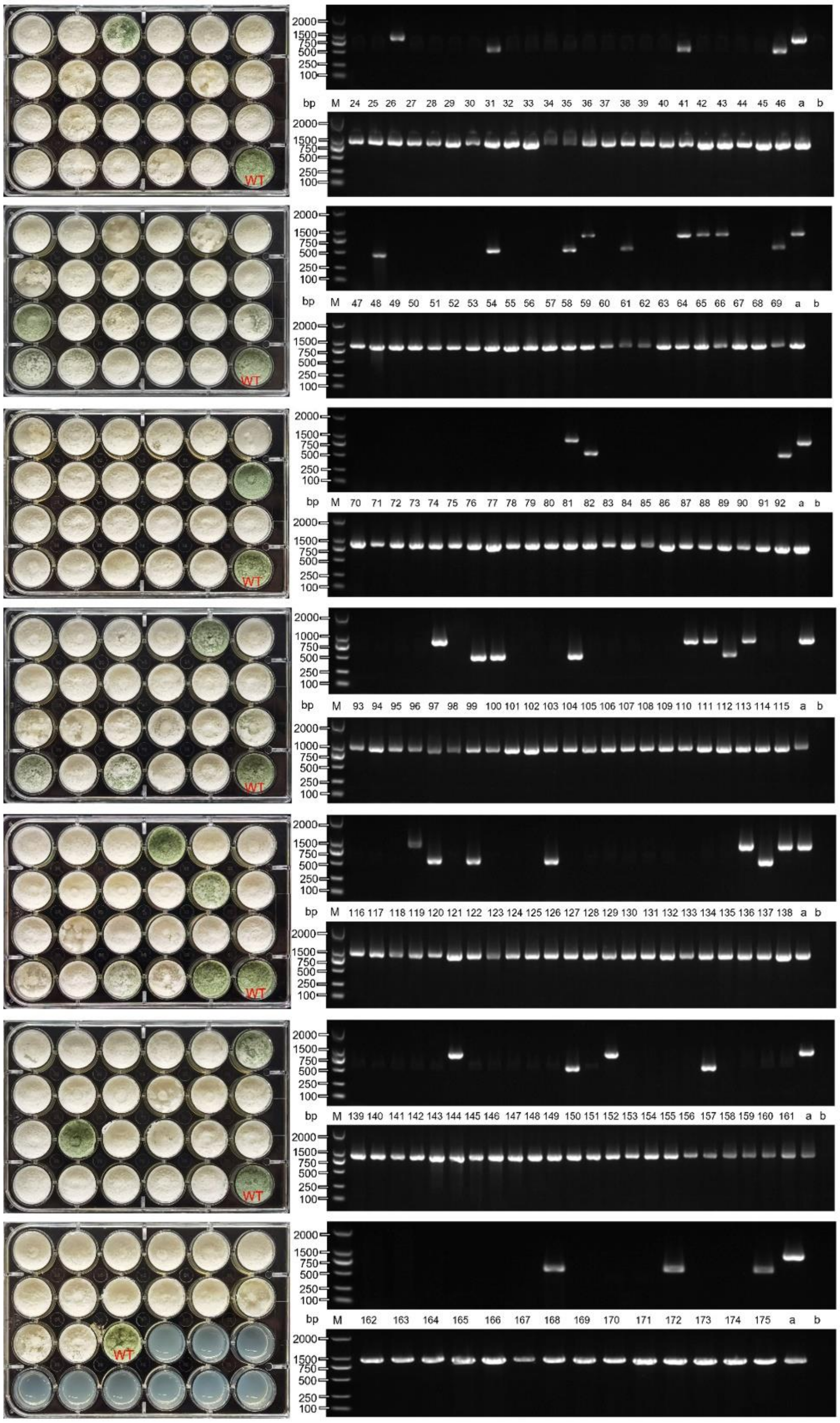

Figure S6. All transformer identification and knockdown efficiency statistics.

The phenotype of transformants on 24 well culture plate and the last well of each 24-well culture plate was inoculated with the T21 wild-type as a control, which is labeled with the red letters WT. The two electropherograms represent the *Thpks1* gene knockout identification and template validation of the transformants, respectively. In each electropherogram, lane a represents the T21 wild type and serves as a positive control, and lane b represents a negative control. The phenotypic identification and molecular identification of the transformants in the above figure correspond to each other.

Table S1 List of oligonucleotides used in this study

Primer name	Sequence	Usage
ITS1	TCCGTAGGTGAACCTGCGG	Identification of T21 Strain
ITS4	TCCTCCGCTTATTGATATGC	Identification of T21 Strain
tef1α-f	CATCGAGAAGTTCGAGAAGG	Identification of T21 Strain
tef1α-r	TACTTGAAGGAACCCTTACC	Identification of T21 Strain
rpb2-f	TGGGWGAYCARAARAAGG	Identification of T21 Strain
rpb2-r	CCCATKGCTTGTTTRCCCATGGC	Identification of T21 Strain
Thpyr4-up-f	aaacgacggccagtgaattcCTACAGTAGTGCCTGCTGGAGCC	Construction of Thpyr4 knockout vector
Thpyr4-up-r	tcagtacaatcGGTGCCAACAGTGACCGATG	Construction of Thpyr4 knockout vector
Thpyr4-gfp-f	gttggcaccGATTGTACTGAGAGTGCACCATATGG	Construction of Thpyr4 knockout vector
Thpyr4-gfp-r	ctcgattcctgGCCATTCAGGCTGCGCAA	Construction of Thpyr4 knockout vector
Thpyr4-down-f	ctgaatggcCAGGAATCGAGGAGGCACC	Construction of Thpyr4 knockout vector
Thpyr4-down-r	accatgattacgccaagcttCGACCATCGACTGCAACCTC	Construction of Thpyr4 knockout vector
(Thpyr4-f)	GTGCTCAAGACACACTACGAC	Identification of Thpyr4 knockout vector and transformants
(Thpyr4-r)	ATGCCTCTACCTACAATGGC	Identification of Thpyr4 knockout vector and transformants
Thpks1-up-f	aaacgacggccagtgaattcCTGATCCACGCTACTTCGGATT	Construction of Thpks1 knockout vector
Thpks1-up-r	cagtacaatcGAGGCTGAACAATCTGACCCTG	Construction of Thpks1 knockout vector
Thpks1-gfp-f	gttcagcctcGATTGTACTGAGAGTGCACCATATGG	Construction of Thpks1 knockout vector
Thpks1-gfp-r	gtcaaaccaagGCCATTCAGGCTGCGCAA	Construction of Thpks1 knockout vector
Thpks1-down-f	ctgaatggcCTTGGTTTGACCGCGCTTG	Construction of Thpks1knockout vector
Thpks1-down-r	accatgattacgccaagcttACCGTTCGTGGTGGACTGTATG	Construction of Thpks1 knockout vector
(Thpks1-f)	GAAGCTTACCATGCGGTGTC	Identification of Thpks1 knockout vector and transformants
(Thpks1-r)	TACCATTGGACCTACGGGATG	Identification of Thpks1 knockout vector and transformants

Cas9-gpda-f	aatgcgtcgagatgaagatctGCCATTCAGGCTGCGCAA	Construction of Thpyr4 and Thpks1 CRISPR/Cas9 vector
Cas9-gpda-r	aagacactgcggGGTGATGTCTGCTCAAGCGG	Construction of Thpyr4 and Thpks1 CRISPR/Cas9 vector
pmv-f	acatcaccCCGCAGTGTCTTGCGTCTCT	Construction of Thpyr4 and Thpks1 CRISPR/Cas9 vector
pmv-r	taagtTTGGCAGTGACTCCGTCTCTG	Construction of Thpyr4 and Thpks1 CRISPR/Cas9 vector
Cas9-trpc-f	cggagtcactgccaaACTTAACGTTACTGAAATCATCAAACAG	Construction of Thpyr4 and Thpks1 CRISPR/Cas9 vector
Cas9-trpc-r	cgcagcctgaatggcgatatcAAGAAGGATTACCTCTAAACAAGTGTACC	Construction of Thpyr4 and Thpks1 CRISPR/Cas9 vector
（Gpda-f）	TAAGCGAAGGAGAATGTGAAGCC	Identification of Thpyr4 and Thpks1 CRISPR/Cas9 vector
（Trpc-r）	CTGGAAGAGGTAAACCCGAAACG	Identification of Thpyr4 and Thpks1 CRISPR/Cas9 vector
actin-f	TCGTGACATCAAGGAGAAG	reference
actin-r	TCAAGACCAAGGACAGAAG	reference
Q-3839-f	CCGCATTTGAAGCCTTTGAG	qPCR Analysis
Q-3839-r	CAGGGAACCTGAGTTTCCTGTG	qPCR Analysis
Q-4143-f	ATCTCGCCGAAAGAGGCTGA	qPCR Analysis
Q-4143-r	CTCGCTGGGTTGATGGTGTC	qPCR Analysis
Q-6889-f	ATGCGAGACGTTGGCAAATC	qPCR Analysis
Q-6889-r	ACTTGACTACGGCAGCCTTGA	qPCR Analysis
RT-cas9-f	CTACAAGGTCTACGACGTCC	RT-PCR Analysis
RT-cas9-r	TCGCTGATCAGCTTCTGTTC	RT-PCR Analysis

Table S2 Genome assembly and annotation statistics for *T. hamatum* T21 genomes.

Indicators of genome assembly	T21
length of genome assembly (Mb)	42.05
number of contigs	251
N50 of contigs (Mb)	2.04
Total length of retrotransposons (Mb)	16.17
Number of annotated genes	8,170
Average CDS length (bp)	1470
Average protein length (bp)	490
